# Artificial intelligence in renal pathology: Current status and future

**DOI:** 10.17305/bjbms.2022.8318

**Published:** 2023-03-16

**Authors:** Chunyue Feng, Fei Liu

**Affiliations:** 1 Department of Nephrology, The Children’s Hospital, Zhejiang University School of Medicine, Hangzhou, China; 2 National Clinical Research Center for Child Health, Hangzhou, China

**Keywords:** Artificial intelligence (AI), deep learning (DL), digital pathology, nephropathology, image analysis, convolutional neural networks (CNNs)

## Abstract

Renal biopsy pathology is an essential gold standard for the diagnosis of most kidney diseases. With the increase in the incidence rate of kidney diseases, the lack of renal pathologists, and an imbalance in their distribution, there is an urgent need for a new renal pathological diagnosis model. Advances in artificial intelligence (AI) along with the growing digitization of pathology slides for diagnosis are promising approach to meet the demand for more accurate detection, classification, and prediction of the outcome of renal pathology. AI has contributed substantially to a variety of clinical applications, including renal pathology. Deep learning, a subfield of AI that is highly flexible and supports automatic feature extraction, is increasingly being used in multiple areas of pathology. In this narrative review, we first provide a general description of AI methods, and then discuss the current and prospective applications of AI in the field of renal pathology. Both diagnostic and predictive prognostic applications are covered, emphasizing AI in renal pathology images, predictive models, and 3D in renal pathology. Finally, we outline the challenges associated with the implementation of AI platforms in renal pathology and provide our perspective on how these platforms might change in this field.

## Introduction

Renal biopsy pathology is an essential gold standard for the diagnosis of various glomerular and tubular diseases. Renal pathologists work closely with nephrologists to evaluate renal biopsy through light microscopy, immunofluorescence microscopy, electron microscopy, and clinical information to render a definitive clinicopathological diagnosis [1, 2]. However, the pathology of renal biopsy has long lacked a unified and standardized diagnostic model. The level of renal biopsy specimen preparation and pathological slice observation in different hospitals is uneven, and there is considerable variation among different observers. Poor reproducibility seriously affects the diagnosis of renal biopsy [3, 4]. It not only delays the treatment of patients but also is not conducive to clinical research and academic communication. The incidence rate of kidney disease is rising year by year, and the traditional diagnostic method of renal biopsy is far behind the needs, so it is urgent to create new technologies for a more accurate diagnosis [5, 6].

With the fast growing of the computer science and technology field, artificial intelligence (AI) has made the development and application of medical expert systems and artificial neural networks a reality, and this field has made significant progress and breakthroughs. Whole-slide images (WSIs) are generated by high-throughput digitization slides, which makes it possible to effectively use computer-assisted histopathologic analysis [3, 7]. Deep learning (DL) is a machine learning (ML) algorithm that uses multiple layers to progressively extract higher-level features from the original input. Recently, DL architectures have shown significant potential for image segmentation, reconstruction, recognition, and classification. Moreover, AI is easy to quantify and standardize, making it an assistant for standardized quantification and a bridge between clinics and pathology. AI can potentially effectively reduce misdiagnosis and improve the accuracy of the pathological diagnosis of renal disease.

In the last several decades, DL has defined many of the technology applications for computer vision tasks and is used in many aspects of healthcare, due to its advances in algorithms, more accessible computing power, and the ability to manage large datasets [8–10]. Digital pathology was initially used for research purposes but has been increasingly employed as a clinical tool for imaging and processing pathological slides. Currently, combined efforts have been made by computer scientists and pathologists to apply the latest AI techniques to the analysis of pathological slides for diagnosis, prediction of prognosis, treatment, as well as other clinically related purposes, such as improving the efficiency of the diagnostic workflow [11–13]. Renal pathology can also benefit from this digital pathology with a powerful DL algorithm.

In this narrative review, we first give a general description of AI methods. It is followed by covering the current and prospective applications of AI in renal pathology, including both diagnostic and predictive prognostic applications, with emphasis on the role of AI in renal pathology images, predictive models, and 3D in renal pathology. Finally, we outline the limitations and challenges associated with implementing AI platforms in renal pathology and provide our perspective on how these platforms might change in this field.

## A brief overview of AI

AI aims to design computer systems that simulate human intelligence. ML is a subdivision of AI that combines algorithms and statistical models trained on sample data. Through case learning, it can identify patterns and then make predictions from new data [14]. Predictions can be categorized as learning from humans or identifying unknown signals. For the former, a model is generated from human-annotated datasets learning to help pathologists complete the diagnostic task in the clinical workflow. For the latter, a model can be developed using the same input data and labels based on the outcome. The two types can also be combined in applications, such as models that identify morphological features as building blocks followed by the prediction of outcomes. As a special kind of artificial neural network (ANN), DL is a category of ML algorithms. Inspired by biological neural networks, DL and other ANNs build a network model with multiple connected layers in a mathematical way. The first network layer, known as the input layer, receives and inputs from slide images. Using a set of parameters, the outputs can be calculated automatically. The next network layer receives inputs from its previous layers, which also uses parameters and calculates the outputs. Finally, the last network layer is the output layer, which calculates the outputs of the whole model. The layers connecting the input and output layers are called the hidden layers, because they are invisible and do not receive model input or generate model outputs directly. Due to the progress of computational hardware, the scale of data accumulation, and the improvement in algorithms, hundreds of layers can be built in a popular ANN [15].

Currently, the convolutional neural network (CNN) is the most popular DL technology. Because of its unique characteristics in nuclear detection and histological image classification and segmentation, it has been widely used in pathology. A CNN with a series of convolutional layers as the hidden layers can make the network deeper. This structure of the network can extract representative features for prediction. As a DL method, CNN combines the optimization of a set of feature extraction filters with related classification problems. CNN can produce a set of feature extraction filters that have been optimized to provide feature values for nephropathological situations. CNN-based methods have been applied to detect image-based information and identify, quantify cell and histological features as segmentation tasks [16]. Recurrent neural network (RNN) is another commonly used DL type. A RNN is characterized by a circular connection, allowing information to flow back and be stored in its hidden layers. Therefore, the previous outputs can influence the current inputs and outputs [17].

AI training algorithms can be divided into supervised, semi-supervised, and unsupervised learning approaches depending on the labels. The algorithm receives a group of data points as input, and each data point has a related label in supervised learning, whereas in unsupervised learning, the algorithm receives data without a label. Semi-supervised learning is the combination of both approaches: the algorithm receives a set of data points, but only a subset of these points has associated labels [18]. Semi-supervised and unsupervised learning algorithms enable the computer to explore the natural patterns in the data without the interference from a predefined output set [19]. Thus, AI-assisted technology can facilitate the discovery of new features and help to reveal potential relevance in the field of renal pathology.

**Figure 1.**
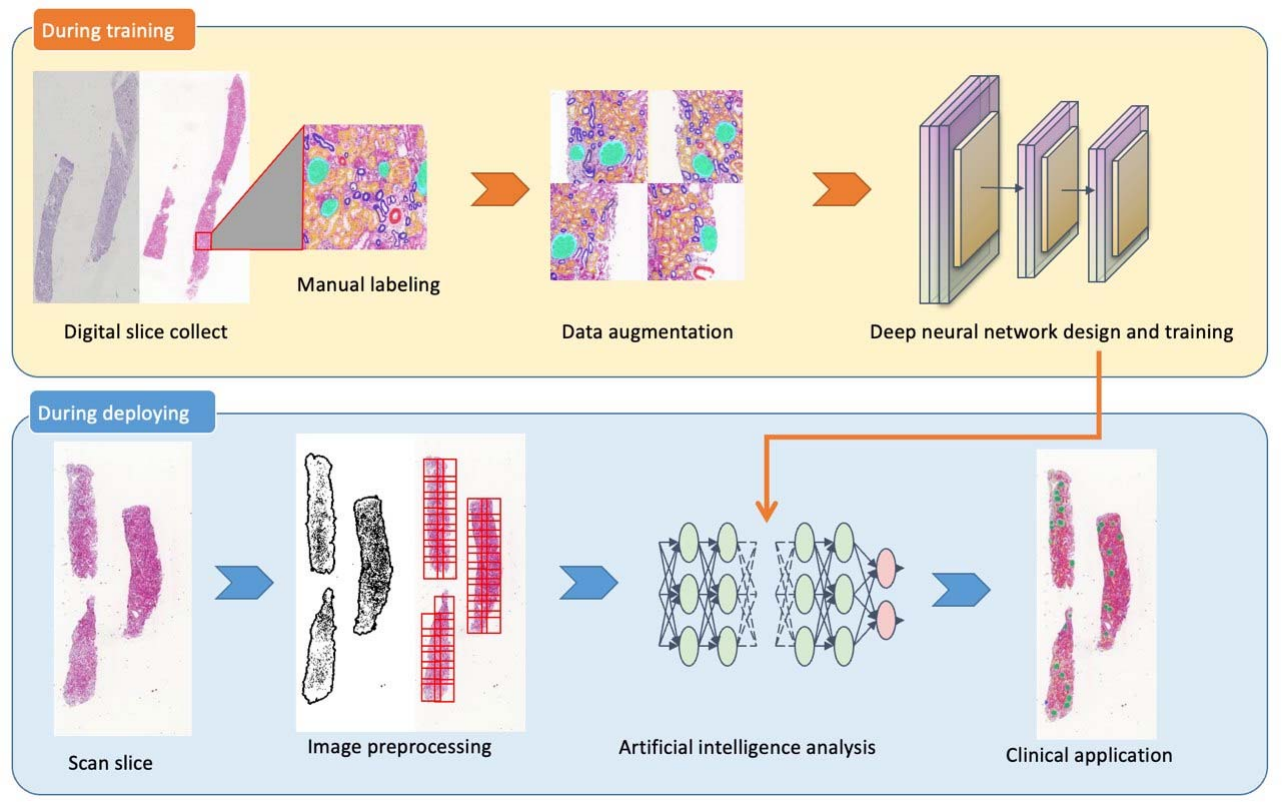
Flowcharts of artificial intelligence technology during training and deployment.

Pathology image segmentation based on DL is generally divided into the following steps: data preparation, image preprocessing (image normalization and augmentation), selection and construction of model (software and model selection and training phase), postprocessing, feature extraction, and association with disease. Figure 1 illustrates the flowcharts of a segmentation neural network during training and during deployment in practical work.

## AI application in renal pathology

### Pathology image analysis using DL algorithms

The study of pathological image analysis based on AI has been a hot spot in pathology in recent years. With the rapid development of medical imaging techniques, such as image scanning technology and visualization software, WSI has become a conventional diagnostic method. Many ML algorithms, including CNNs, have been promoted to segment pathology images automatically [20]. Thus, pathology image segmentation based on DL has played a crucial role in WSI analysis. In recent years, DL algorithms have shown excellent application prospects in pathology image analysis, for example, tumor region identification and metastasis detection in oncology [9, 10, 15, 21, 22]. The research and application of AI in oncology have been quite mature, and many methods have been applied in the clinic for precision oncology, such as improving diagnostic accuracy and identifying new biomarkers. The development of AI in oncology brings inspiration and hope for AI in the pathology in other disciplines, including renal pathology.

In renal biopsy pathology, an extensive selection of staining methods is used in specimen processing, necessitating a multifaceted evaluation. Morphological evaluation depends on the slices quality, the expertise of pathologist to identify individual structures and related changes, and their use of quantitative or semiquantitative indicators to evaluate the degree of tissue damage. Quantitative evaluation of visual histology, such as the counting, morphometry, and distribution analysis of some histological primordia, can be a powerful predictor of the prognosis for various kidney diseases. However, quantitative analysis with the human eye remains a challenge. The implementation of automatic image analysis tools and their integration into the pathological workflow can largely facilitate the quantitative evaluation of renal biopsy specimens. The DL segmentation tool can greatly enhance the derivation of both visual and subvisual morphological organization features (such as shape, texture, and graphic features), which is related to the diagnosis and results.

In recent years, accumulating reports showing nephropathology diagnosis have been based on DL. Initially, these studies were mainly focused on the glomerulus. The first attempt was to determine the glomerular pathology [23–33], with models to distinguish between sclerotic and nonsclerotic glomeruli reported later [34–42]. The application of automated methods to measure the percentage of sclerotic glomeruli can reduce the variability of assessment, increase throughput, and reduce the burden on pathologists. Based on the use of DL, Barros et al. [43] classified proliferative glomerular lesions, and Chagas et al. [44] refined the classification of glomerular hypercellularity, including mesangial, endocapillary, and both lesions. Sheehan et al. also obtained precise findings for glomerulus segmentation, quantified mesangial matrix proliferation, numbers of nuclei, and glomerular tuft [45, 46]. In addition to the glomerulus, segmentation of the other renal basic structures has been reported. For example, Hermsen et al. presented a CNN for multiclass segmentation, including glomeruli, tubules, and interstitium of nephrectomy samples stained with PAS in transplant biopsies. Their work is the first validation of quantitative results obtained by CNN with the Banff classification system [47]. Jayapandian et al. developed and evaluated the DL-based segmentation of renal cortex tissue structure, including HE, PAS, silver, and trichrome staining, divided into training, validation, and testing sets. Finally, they found that PAS-stained WSIs can yield the best consistency between pathologists and DL segmentation of all structures, such as glomerular tufts, glomerular tuft plus Bowman’s capsule, proximal tubules, distal tubules, peritubular capillaries, arteries, and afferent arterioles [48, 49]. Ginley et al. applied modern ML tools for automated computational detection of multiple renal pathologies, including interstitial fibrosis, tubular atrophy, and glomerulosclerosis. This application provides a standardized method for assessing the severity of chronic kidney injury [50, 51]. Uchino et al. used DL to classify seven main glomerular pathological manifestations, including global sclerosis, segmental sclerosis, endocapillary proliferation, mesangial cell proliferation, mesangial matrix accumulation, and crescent and basement membrane structure changes [52]. Zeng et al. established DL algorithms to locate glomeruli, identify glomerular lesions, and classify and quantify different types of intrinsic glomerular cells. They also generated a network-based mesangial hypercellularity score in PAS stain slides in 400 Chinese patients with immunoglobulin A nephropathy (IgAN) [53]. Bouteldja et al. developed a DL algorithm for accurate multiclass segmentation of digital WSIs of PAS-stained kidneys from multiple experimental renal pathology models (e.g., unilateral ureteral obstruction, ischaemia reperfusion injury, and adenine models) of various species, including mice, rats, pigs, marmosets, and bears [54]. Most recently, the prognostic features of kidney histology were automatically obtained by segmentation of the subcomponents of glomerular volume, glomerular density, interstitial fibrosis, tubular atrophy, and vascular intimal thickness [55]. Another recent approach is to detect and quantify interstitial, tubular, and mononuclear leukocyte infiltration in both preimplantation and posttransplant biopsies for risk stratification of allografts and posttransplantation monitoring in clinical practice [56]. Weis et al. investigated the potential value of applying ML in the automatized recognition of common glomerular morphological changes, such as amyloidosis, nodular sclerosis, global sclerosis, mesangial hypercellularity, etc. [57]. Theilig et al. focused on the renal tubules, distinguished between normal and abnormal renal tubules, and thus contributed to a comprehensive understanding of renal pathological structure [58].

In addition to the application of AI in light microscopy, Ligabue et al. investigated the classification accuracy of kidney biopsy direct immunofluorescence through CNN. They also described the specimens in terms of appearance, distribution, location, and intensity of the glomerular deposits stained with fluorescent antibodies against IgG, IgA, IgM, C1q, and C3 complement fractions, fibrinogen, as well as κ- and λ-light chains [59]. Furthermore, Zhang et al. segmented and classified the deposition patterns in immunofluorescence images of glomerular objects, including granules on the capillary wall, lumps on the capillary wall, linear on the capillary wall, granules in the mesangial region, and lumps in the mesangial region [60]. In light of immunofluorescence pathology, AI diagnosis of renal pathology is more comprehensive. It is believed that the combination of immunofluorescence microscopy and light microscopy data can be realized soon. All of the above attempts by different teams provide the basis for the automatic diagnosis of pathological images. Using AI to automatically identify special renal pathologies can reduce the workload of pathologists. In addition, the quantitative evaluation of computer technology is objective and can reduce variability between different pathologists. Table 1 characterizes the datasets used in the development of AI and the abovementioned reference methods.

**Table 1:** Current applications of AI in nephropathology

**Author**	**Year**	**Methods**	**Imaging**	**Species**	**Tasks**
Kato et al.	2015	HOG+SVM	IHC	Rat	Detection (glomeruli)
Maree et al.	2016	Icy and Cytomine	TRI	Human	Detection (glomeruli)
Sarder et al.	2016	Color deconvolution, Gabor filtering, bottleneck detection	HE, PAS	Rat	Segmentation (glomeruli)
Temerinac-Ott et al.	2017	CNN	Jones HE, PAS, Sirius red, CD10	Human	Detection (glomeruli)
Gadermayr et al.	2017	U-Net	PAS	Mouse	Detection and segmentation (glomeruli)
Barros et al.	2017	PathoSpotter-K kNN	PAS, HE	Human	Classification (proliferative glomerular lesions)
Marsh et al.	2018	CNN	Frozen HE	Human	Classification and segmentation (glomeruli)
Gallego et al.	2018	CNN	PAS	Human	Classification and detection (glomeruli)
Bukowy et al.	2018	Faster RCNN	TRI	Rat/human	Classification (glomeruli)
Kolachalama et al.	2018	CNN	TRI	Human	Classification (interstitial fibrosis)
Simon et al.	2018	LBP+SVM	HE, PAS, SIL, TRI	Mouse, rat, human	Detection (glomeruli)
Sheehan et al.	2018 & 2019	DNNs	PAS	Mouse	Segmentation (glomeruli); quantification (mesangial matrix proliferation, numbers of nuclei, and glomerular tuft).
Kannan et al.	2019	Inception V3 CNN	TRI	Human	Detection and segmentation (globally sclerosed glomeruli)
Hermsen et al.	2019	CNN	PAS	Human	Segmentation (glomeruli, tubuli, and interstitium)
Ginley et al.	2019	CNN	PAS	Human	Quantification (glomeruli: nuclei, capillary lumina, and Bowman spaces)
Bueno et al.	2020	CNN/SegNet	PAS	Human	Classification, segmentation (normal/sclerosed glomeruli)
Zeng et al.	2020	CNN	PAS	Human	Identification and classification (glomeruli, intrinsic glomerular cell recognition)
Uchino et al.	2020	InceptionV3-CNN	PAS, SIL	Human	Classification (seven glomerular pathological findings)
Ligabue et al.	2020	CNN	IF	Human	Identification (IgG, IgA, IgM, C1q, and C3 complement fractions, fibrinogen, and κ- and λ-light chains)
Chagas et al.	2020	CNN	HE, PAS	Human	Classification (mesangial and endocapilar hypercellularity)
Cordido et al.	2020	CystAnalyser	HE	Mice	Detection and quantification (cysts in polycystic kidney)
Marsh et al.	2021	VGG16	Frozen HE	Human	Quantification (glomerulosclerosis)
Jayapandian et al.	2021	U-Net	HE, PAS, SIL,TRI	Human	Detection, segmentation, and quantification (glomeruli, tubuli, capillaries, arteries)
Bouteldja et al.	2021	CNN	PAS	Mouse, rats, pigs, marmosets, bears	Segmentation (different experimental disease models)
Gallego et al.	2021	U-Net	PAS, HE	Human	Segmentation, classification, and quantification (glomerulosclerosis)
Ginley et al.	2021	CNN	PAS	Human	Detection and segmentation (interstitial fibrosis, tubular atrophy, glomerulosclerosis)
Zhang et al.	2022	U-Net	IF	Human	Detection (glomeruli deposition pattern in IF)
Yi et al.	2022	R-CNN	PAS	Human	Quantification (intersitium, tubules, mononuclear leukocyte infiltration)
Marechal et al.	2022	CNN	Masson	Human	Identification (glomerular volume, glomerular density, interstitial fibrosis, tubular atrophy, vascular intimal thickness)
Weis et al.	2022	CNN	PAS	Human	Classification (glomerular structural changes)
Pesce et al.	2022	IBM Watson	PAS	Human	Identification (sclerotic and non-sclerotic glomeruli)
Hara et al.	2022	U-Net	PAS	Human	Classification (normal and abnormal tubules)

AI: Artificial intelligence; CNN: Convolutional neural network; HOG: Histogram of oriented gradients; SVM: Support vector machine; LBP: Local binary patterns; FCNN: Fully convolutional network; kNN: k-nearest neighbor; R-CNN: Region-based convolutional neural network; IHC: Immunohistochemistry; HE: Hematoxylin eosin; TRI: Trichrome Masson; PAS: Periodic acid-Schiff; SIL: Periodic acid-methenamine silver; IF: Immunofluorescence.

Kidney transplant pathology includes the evaluation of donors’ transplanted kidney before implantation and the evaluation of recipients after transplantation. Preimplantation kidney biopsy is helpful to determine the suitability of the kidney organ as a transplant, which is often performed in an urgent situation. If an on-call pathologist without specific expertise needs to evaluate the adequacy of the biopsy, they will evaluate the presence of glomerular damage based on the percentage of sclerotic glomeruli to help guiding the decision regarding the adaptation and allocation or discarding of organ [61]. In this case, specialized pathologists are more reproducible and reliable than general pathologists [62]. The Web platform lays the foundation for the introduction of AI in the field of transplantation, helping to create new diagnostic algorithms and tools with the aim of increasing the precision of organ assessment and improve its predictive value for the prognosis of the transplant [63]. These studies mainly focus on the detection and classification of glomerular structures based on AI [34, 35, 64, 65]. In contrast, there are a few studies on tubular and vascular structures [66, 67]. Moreover, posttransplant renal pathology needs to work on the evaluation of rejection as well as functional alterations of posttransplant. Detailed criteria for biopsy assessment have been established, such as the Banff criteria, which require specific training and expertise. Most studies applying automated algorithms to renal biopsy mainly quantify inflammatory elements or interstitial fibrosis related to rejection [68, 69].

AI is also involved in nephrooncology. Renal cell carcinoma (RCC) is the most common renal cancer, and the histopathologic classification of RCC is crucial to the diagnosis, prognosis, and management of patients. First, the spatial distribution of nuclear size is calculated through automatic WSI analysis, which can be used to localize these tumor regions and distinguish between high-grade and low-grade tumors, thus facilitating the detection of clear cell RCC [70]. Recently, Fenstermaker et al. developed and validated a CNN that can identify tumor grade and histologic subtypes of RCC, including clear cell RCC, papillary RCC, and chromophobe RCC [71]. In addition to the three RCC subtypes, Zhu et al. developed an AI model that also includes benign tumors or oncocytoma [72]. Moreover, Tabibu et al. classified RCC subtypes and further extracted characteristics for survival prediction [73]. Marostica et al. developed an automated method to predict the subtypes, prognosis, and genomic aberrations of patients with RCC using histopathology images, which could provide information for treatment decisions [74].

### Predictive models for prognosis

Focusing on recognition problems, such as detection and segmentation, as mentioned above, may be low-level tasks for AI. Higher-level tasks are to use algorithms to predict the risk of kidney diseases and evaluate the treatment response. AI does not simply classify images but also potentially can identify aggressive imaging phenotypes based on their similar molecular subtypes. There is an opportunity to combine the advances in molecular and imaging risk stratification to provide a deeper understanding of renal pathology and to identify new therapeutic targets.

Mariani et al. demonstrated that interstitial fibrosis scored based on the WSI of kidney biopsies can be used in the prognosis of proteinuric glomerulopathies. They found that the degree of interstitial fibrosis in different types of proteinuric glomerulopathy was associated with reduced risk of estimated glomerular filtration rate and with the expression of inflammatory and fibrotic genes and may have predictive value in assessing the risk of progression [75]. Chen et al. established and validated the Nanjing IgAN Risk Stratification System by applying a gradient tree boosting method. They selected the 10 most important variables from 36 candidate variables and then built a simplified scoring scale model by stepwise Cox regression analysis based on these 10 variables. A prediction model using routinely available characteristics and based on the combination of a ML algorithm and survival analysis can stratify risk for the progression of kidney disease in the setting of IgAN. This system can serve as a more favorable tool to strengthen individualized treatment and management in patients with IgAN [76].

AI-based algorithms can provide an objective, accurate, and effective evaluation and economical method for clinical trials. If a model can better predict the life expectancy of a given patient than the existing risk stratification systems, it is expected to be widely used. With the application of AI technology, the field of predictive models for prognosis has developed rapidly.

### 3D digital pathology

Although current AI applications in renal pathology are mainly for image analysis, they also have significant potential use in other areas. A more recently studied application is the overlay of multiple 2D slides to reconstruct a 3D volume. This allows the pathologist to evaluate the entire resected tissue or biopsy. 3D reconstruction can provide better insight into architectural features and spatial arrangements with other structures [77]. The addition of a third dimension to the flat images of organs, tumors, and resected margins can open up a different understanding of pathology. High-quality WSI builds the base for successful 3D reconstruction. Even a tissue slice with a “flat” surface has a three-dimensional aspect: some diagnostic problems need to focus on several layers on a slide and use micrometer adjustment to simulate microscopic evaluation. On a digital image, this “depth” or z-stack dimension of first-view “flat” images is highlighted [78].

Klingberg et al. counted all individual glomeruli in mouse kidneys and determined the size of capillary tufts by combining fluorescence labeling of endothelial cells in vivo, a novel tissue clearance technique, light sheet fluorescence microscopy, and image analysis automated registration, which can reconstruct the whole kidney in three dimensions [79]. Uesugi et al. described a new technology for visualizing the 3D structure of the human kidney microvasculature. This technology is based on a virtual slide system with double immunostaining on a series of sections. This study demonstrates the 3D imaging of human renal microvasculature for the first time, including glomeruli, interlobular arteries, arterioles, and peritubular capillaries. This method advantages the field by providing detailed images of the microcirculation thereby facilitating our understanding of the development of renal injury [80]. Moreover, Chen et al. demonstrated 3D pathology at high spectral resolution with infrared spectromicroscopy. This study brings a quantitative evaluation on the pathological effects on the small functional units of the kidney. 3D chemical imaging using infrared spectromicroscopy is a new method that can determine the pathophysiological status of small functional units in vital organs, such as kidneys, to provide more robust information for anatomic pathology [81].

If the 2D WSI serial sections can be applied to holistically quantify the 3D context of glomeruli, this technique will robustly improve the reproducibility of glomerular phenotype analyzed by pathologists. The advantages of 3D quantitative analysis are illustrated by using advanced microscopy imaging techniques, such as confocal microscopy, electron microscopy, and automated tape-collecting microtomes. Although the current research on 3D in the kidney is limited, clinical applications of this technology are expected to be developed in the future.

### Integration of AI with other clinical data

Histopathologic image analysis is not limited to visual analysis but also needs to include several other sources of data coming from patient demographic information, medical history, laboratory results, and other clinical records. The technology of natural language processing can be employed to extract relevant information and link this information with histopathologic findings. Natural language processing also began to benefit from DL based on AI technology. AI is essential for filtering the information from these different sources, thereby helping pathologists make the best clinical decisions for patients. AI is potentiated to discover more complicated or subtle connections which humans would not find. AI can combine the images with clinical and outcome data to achieve high-dimensional analysis, which is beyond the capacity of the human brain to complete alone. Abundant data sources can transform pathology from a clinical science to an informatics science in which the tissue would be only one of the data sources [82].

The purpose of combining pathology with omics or other computational methods depends on the development of reliable image registration methods. With the advent of DL, substantial progress is expected to be made in this area in the near future. Diseases with molecular characteristics can be detected by genomics, epigenomics, proteomics, and metabolomics analysis. The subvisual signals detectable by AI can be used to evaluate and support morphologic changes, which can be associated with these molecular features to achieve more accurate, comprehensive, and clinically relevant pathology diagnosis/classification.

## Limitations of AI for implementation in renal pathology

Although AI in renal pathology has broad application prospects, its effective implementation in clinical practice still faces many challenges. First, the research datasets currently commonly used are small and come from single institutions, which leads to the potential for overfitting algorithms [83]. This can only be overcome by linking data from multiple sources, including clinical information and genetic profiles. Collaboration between multiple healthcare fields with secure and interoperable data flows can also help expand the data resource and improve the algorithms. Second, AI lacks clear interpretable parameters that can help researchers and clinicians to determine whether the model is making reasonable decisions. The problem of “black box” in AI is significant in the medical field, making it difficult to guarantee consistent performance. Third, when AI enters the field of renal pathology, it must adapt to ethical and safety issues. As the current laws and regulations are not enough to solve these problems, the privacy of patients, data security, and data ownership are major issues. As mentioned above, an international consensus on the ethics and safety use of AI in renal pathology needs to be reached by society as a whole [12]. AI for renal pathology is a new industry, and with deeper application, more disadvantages will emerge, requiring the joint efforts of the whole society.

## Conclusion and future perspective

The application of AI in the field of renal pathology is increasing, and AI methods have the ability to recognize patterns and combine the information in ways that humans cannot, showing excellent prospects for pathology and precision medicine in the future. An ideal renal pathology diagnosis system based on AI will combine all relevant multimodal imaging data, clinical information, and molecular markers to make an accurate prediction and conform to precise medical movement. AI can not only supplement the work of renal pathologists, reduce their workload, and improve their diagnostic accuracy but also provide more information. Understanding the functions, advantages, and limitations of AI will become crucial for nephropathologists. With the development of AI, pathology is rapidly moving to digital methods. Like any other new innovative technology, the possibilities of development may be beyond the current imagination.
